# Assessment of Environmental Risk Factors for Gestational Diabetes Mellitus: A Ten-Year Systematic Review and Meta-Analysis

**DOI:** 10.3390/jcm14051646

**Published:** 2025-02-28

**Authors:** Sophia Tsokkou, Stefanos-Timoleon Tzintros, Ioannis Konstantinidis, Antonios Keramas, Maria-Nefeli Georgaki, Eleni Stamoula, Alkis Matsas

**Affiliations:** 1 Department of Medicine, Faculty of Health Sciences, Aristotle University of Thessaloniki, 54124 Thessaloniki, Greece; stefanostzintros@gmail.com (S.-T.T.); ikonsc@auth.gr (I.K.); antonios@auth.gr (A.K.); 2 Environmental Engineering Laboratory, Department of Chemical Engineering, Aristotle University of Thessaloniki, 54124 Thessaloniki, Greece; mgeorgaki@cheng.auth.gr; 3 Department of Clinical Pharmacology, School of Medicine, Aristotle University of Thessaloniki, 54124 Thessaloniki, Greece; eleni_stamoula@yahoo.gr; 4 Laboratory of Experimental Surgery and Surgical Research ‘N.S. Christeas’, Medical School, National and Kapodistrian University of Athens, 11527 Athens, Greece

**Keywords:** gestational diabetes mellitus, GDM, fine particulate matter, PM_2.5_, bisphenol A, extreme temperatures, pregnancy

## Abstract

**Background/Objectives**: It is estimated that gestational diabetes mellitus (GDM) affects approximately 14% of pregnant women. This is due to the inability of the body to produce enough insulin for gestation. With greater appearance during the second and third trimesters, GDM has a multifactorial cause including hypertension, cardiovascular issues (CVD), family history both or either type two diabetes mellitus (T2DM) or GDM, obesity, advanced maternal age, and polycystic ovarian syndrome (PCOS). However, it has been suggested that except for genetic predisposition, environmental factors can increase the risk of GDM development to a great extent. The aim of this systematic review and meta-analysis is the examination of different environmental contributors that play a significant role in the development of GDM. **Methods**: The databases used were PubMed and ScienceDirect. The inclusion criteria were a 10-year duration (2014–2024), English language, research articles, and only humans included. Afterwards, tables were created to summarize the most important information from each article. Forest and funnel plots were created to assess the possibility of a greatly significant difference between each environmental contributor. **Results**: Initially, 9361 articles were found. After the automation tools were applied, 706 were left. The total number of articles used in the study after the screening process was 26. Through the systematic review analysis, the following risk factors were stated to play a contributing role with GDM: extreme temperatures (both high and low), organophosphorus flame retardants (OFRs), bisphenol A (BPA), selenium (Se), metallic elements, urinary antimony (Sb), trace elements, thiamine and riboflavin, and fine particulate matter PM_2.5_. **Conclusions**: Through this meta-analysis, it can be concluded that there is statistical significance for fine particulate matter PM_2.5_, especially in the first (*p* < 0.001) and second (*p* < 0.001) trimesters, proving the acknowledged connection between PM_2.5_ and GDM pathogenesis during pregnancy. Apart from that, fetal sex can play an important role in the development of GDM, as there is the greatest risk in males (*p* < 0.001), whereas no correlation between maternal smoking habit and bisphenol A with GDM was found. In conclusion, it can be stated that environmental factors can have a great impact on the development of GDM during the gestational period, but more studies must be carried out to reinforce our outcomes.

## 1. Introduction

### 1.1. Definition and Classification of Diabetes Mellitus

Diabetes mellitus (DM) is characterized as a complex and heterogenous metabolic disorder marked by persistent (chronic) hyperglycemia [1,2,3,4]. Long-term hyperglycemia is a hallmark and a biomarker of DM, and is associated with various microvascular and macrovascular complications, which contribute to the morbidity and mortality of individuals with diabetes [2,4,5]. DM classification is crucial for the study and treatment of the disease. The current classification distinguishes between Type 1 (T1DM); Type 2 (T2DM); specific types of diabetes as a result of other causes, e.g., monogenic diabetes syndrome, drug- or chemical-induced diabetes, and diseases of the exocrine pancreas; and gestational diabetes mellitus (GDM) [6,7].

### 1.2. Definition and Classification of Gestational Diabetes Mellitus

GDM, being one of the most usual complications during pregnancy, was estimated to affect 21.1 million live births, or 16.7%, in 2021 according to the International Diabetes Federation. GDM is frequently defined as hyperglycemia or any degree of glucose intolerance that is diagnosed or develops during pregnancy [8,9,10]. Regardless of the type of treatment or if the diabetes continues after pregnancy, this definition is applicable [9,10]. GDM is categorized into two classes: class A1GDM, which is a milder form and can be managed with diet and lifestyle changes, and class A2GDM, which necessitates pharmacologic treatment of hyperglycemia [8,11].

### 1.3. Gestational Diabetes Mellitus Screening and Diagnosis

During the gestational period, GDM testing is a routine screening process that aims to identify and control affected women early [12]. There have been debates on GDM screening mostly related to the indication of screening, criteria for diagnosis, and timing [13,14]. According to the American Diabetes Association (ADA), women should be screened between the 24th and 28th weeks of gestation, unless they fall in a low-risk category [12,15]. Current diagnostic methods primarily include the oral glucose tolerance test (OGTT) and the Glucose Challenge Test (GCT), usually performed around 24–28 weeks of pregnancy. Recommendations regarding approaches for screening usually include two types: In the first one, pregnant women are screened in a two-step process, involving the measurement of plasma glucose levels 1 h after administration of 50 g glucose. A concentration greater than or equal to 130 mg or 140 mg indicates the need to undergo a 100 gm OGTT on a different day. In the second one, pregnant women are directly tested with a 100 gm OGTT. An established diagnosis is made using the Carpenter and Coustan Criteria [9]. A more current screening approach includes pregnant women undergoing a 75 g OGTT between 24 and 28 weeks of gestation as part of a universal screening protocol. The OGTT involves plasma glucose level measurement after an overnight fast (8 h), followed by oral consumption of 75 g glucose, and plasma glucose measurement at one and two hours. A plasma fasting blood glucose level greater than 126 mg/dL in a pregnant woman is considered as overt diabetes even if no prior history of diabetes exists. GDM is defined as a fasting plasma glucose value greater than 92 mg/dL, a one-hour plasma glucose value greater than 180 mg/dL, or a two-hour plasma glucose value greater than 153 mg/dL. GDM is absent if a pregnant woman has normal values at all three test intervals [16].

### 1.4. Pathophysiology of Gestational Diabetes Mellitus

Various components contribute to the cause of GDM, including hormonal contributors and genetic and environmental factors [14]. Both tissue insulin resistance and β-cell impairment are important elements of the pathogenesis of GDM, which is typically caused by β-cell dysfunction on a background of chronic insulin resistance throughout pregnancy. These impairments typically predate pregnancy and may worsen over time, increasing the risk of T2DM post-pregnancy. Typically, β-cells adapt to the high insulin demands during pregnancy by increasing insulin secretion and β-cell mass. In GDM, however, the β-cells are unable to make up for the insulin resistance, which leads to hyperglycemia [17]. Moreover, β-cell dysfunction is attributable to various mechanisms including glucotoxicity, chronic fuel surplus, and lipotoxicity. These variables hinder the production of insulin, most likely by interfering with important processes such as granule exocytosis, pro-insulin synthesis, glucose sensing and post-translational processing. There is evidence that altered β-cell function in GDM is caused by genetic predisposition, including polymorphisms in genes like KCNQ1 and GCK. Furthermore, the dysfunction is exacerbated by decreased β-cell mass brought on by apoptosis, epigenetic changes (e.g., suppression of PDX1 expression), and decreased β-cell proliferation [17]. Hormones such placental lactogen, progesterone, and cortisol mediate pregnancy-induced insulin resistance, which exacerbates these defects. A vicious cycle of hyperglycemia, increased insulin demand, and further β-cell damage is produced by the interaction of these variables [17].

The aim of this systematic review and meta-analysis is the examination of different environmental contributors that play a significant role in the development of GDM.

## 2. Materials and Methods

### 2.1. Systematic Review and Screening

**Figure 1:**
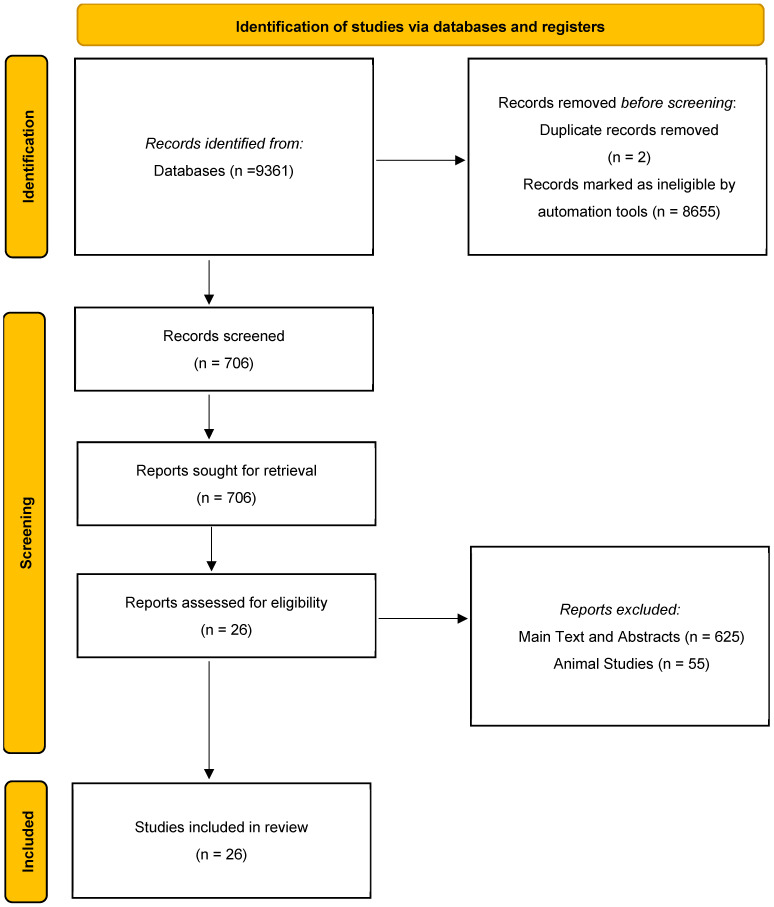
PRISMA—flow diagram [18].

A PRISMA flow diagram (Figure 1) was prepared using the keywords “Gestational Diabetes Mellitus”, “GDM”, “Environmental Risk Factors”, “Social Factors”, and “ERF” to identify the relevant data from databases including PubMed, Scopus, Cochrane, and ScienceDirect, accessed in September 2024. The inclusion criteria were a 10-year duration (2014–2024), English language, research articles, and only humans. The screening process was performed by two independent researchers to eliminate bias and any conflicts were resolved by a third reviewer. A table was prepared, including all relevant articles that were assessed and accepted as appropriate for this systematic review. Data containing statistical data that were considered suitable for the aim of this study were included in the meta-analysis. R Studio 4.4.2 and Jamovi Software 2.6.25 were used to perform the forest plots. The study was not registered in PROSPERO.

### 2.2. Meta-Analysis

For the meta-analysis, both R-studio and Jamovi Software were used for the production of the forest and funnel plots and the statistical assessment for the level of bias and heterogenicity present.

#### 2.2.1. Meta-Analysis in R-Studio Using Odds Ratio (OR) Data

The metafor tool in R Studio was used to construct a forest plot that summarized the 95% CI and odds ratio (OR). In order to account for study heterogeneity, a random-effects meta-analysis model was used. Important metrics such as I^2^, Cochran’s Q, τau^2^, and associated *p*-values were computed. Visual representations of the combined effect size and heterogeneity statistics are provided, together with interpretive notes beneath the graphic.

#### 2.2.2. Meta-Analysis in Jamovi Software for Dichotomous Model (DM) Data

The analysis was carried out using the log odds ratio as the outcome measure. A random-effects model was fitted to the data. The amount of heterogeneity (i.e., tau^2^) was estimated using the restricted maximum-likelihood estimator [19]. In addition to the estimate of tau^2^, the Q-test for heterogeneity [20] and the I^2^ statistic are reported. In case any amount of heterogeneity is detected (i.e., tau^2^ > 0, regardless of the results of the Q-test), a prediction interval for the true outcomes is also provided.

## 3. Results

### 3.1. Systematic Review

Initially, 9361 articles were found. After the automation tools were applied, 706 were left. The articles were screened and assessed for their relevance by two independent observers and any conflicts found were resolved by a third reviewer. Studies with a low level of relevance based on their main text and abstract were excluded. Additionally, any animal studies and studies that were reviews and were not eliminated during the automation tool assessment were excluded at this stage. The total number of articles used in the study after the screening process was 26. A total of 4,088,576 participants were included in the study. The range of age of GDM patients was 27.55 (4.48) to 33.3 (4.9) and non-GDM was 26.33 (4.02) to 33.7 (3.8). The most common exposures women had that increased the likelihood of developing GDM included fine particulate matter (PM_2.5_), extreme temperatures (both extreme high and extreme low), organophosphorus flame retardants, bisphenol A, and metallic and trace elements including urinary nickel (Ni), arsenic (As), cadmium (Cd), antimony (Sb), cobalt (Co), vanadium (V), and selenium (Se) (Table 1, Table 2, Table 3, Table 4 and Table 5).

### 3.2. Meta-Analysis

**Figure 2:**
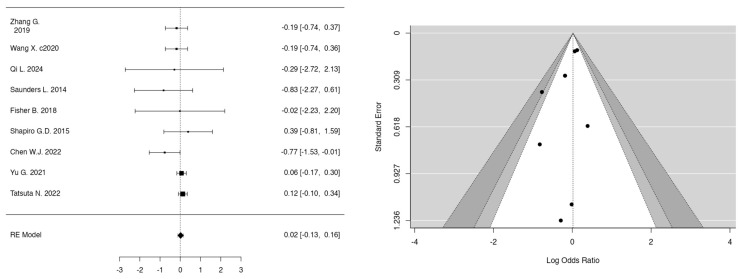
Forest and funnel plots for non-smoking and smoking as risk factors for GDM.

**Figure 3:**
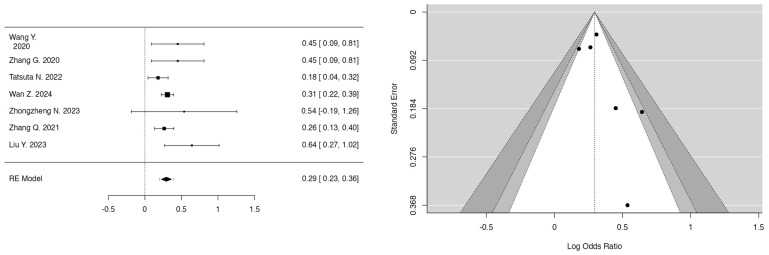
Forest and funnel plots for fetus sex as a risk factor for GDM.

**Figure 4:**
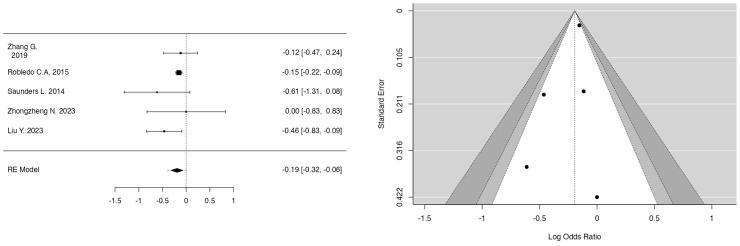
Forest and funnel plots for parity as a risk factor for GDM.

**Figure 5:**
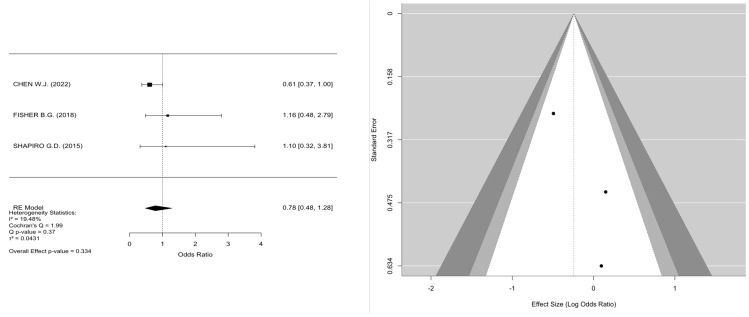
Forest and funnel plots for bisphenol A as a risk factor for GDM.

**Figure 6:**
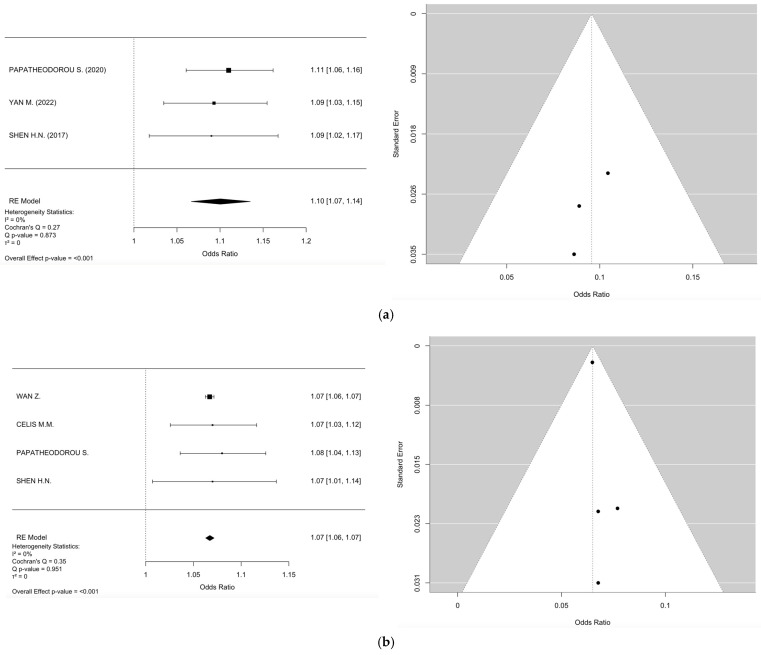
(**a**) Forest and funnel plots for PM_2.5_ as a risk factor for GDM in the 1st trimester. (**b**) Forest and funnel plots for PM_2.5_ as a risk factor for GDM in the 2nd trimester.

The meta-analyses investigated the relationships between smoking, fetal sex, parity, bisphenol A (BPA), and PM_2.5_ in the first and second trimester and the development of GDM. In the case of smoking (Figure 2) (k = 9), the mean log odds ratio was 0.02 (95% CI: −0.13 to 0.16), indicating no significant difference (*p* = 0.8008) or heterogeneity (I^2^ = 0.00%), thus stating no relation of smoking to GDM development. In the analysis of fetal sex (Figure 3) (k = 7), male fetuses exhibited a statistically significant correlation with an elevated risk of GDM (log OR = 0.30, 95% CI: 0.23 to 0.36, *p* < 0.0001, I^2^ = 10.69%). Additionally, parity (Figure 4) (k = 5) demonstrated a significant correlation, indicating that multiparous women had an elevated risk of GDM relative to primiparous women (log OR = −0.19, 95% CI: −0.32 to −0.06, *p* = 0.0033, I^2^ = 14.38%). Moreover, bisphenol A (BPA) exposure (Figure 5) (k = 3) exhibited no significant correlation with GDM (*p* = 0.334, I^2^ = 19.48%), indicating an absence of a relationship with GDM development. Furthermore, PM_2.5_ (Figure 6) levels in both the first trimester (k = 3) and second trimester (k = 4) revealed a significant association for the development of GDM as the pooled odds ratio (OR) for the first trimester was 1.10 (95% CI: 1.07–1.14, *p* < 0.001) and for the second trimester was 1.07 (95% CI: 1.04–1.10, *p* < 0.001) with no significant heterogenicity (I^2^ = 0.00% in both forest plots). In summary, heterogeneity was minimal across all studies, and no significant outliers or biases were identified, nor was any publication bias detected from the funnel plots.

## 4. Discussion

### 4.1. PM_2.5_ and Risk for GDM Development

In 2024, in retrospective study by Wan Z. et al., it was revealed that there are statistically significant differences in the concentrations of air pollutants (PM_2.5_, SO_2_, and O_3_) between non-GDM and GDM groups [21]. It was also discussed that there is a statistically significant correlation between the incidence of GDM and the preconception period, as well as the first and second trimesters [21]. Their findings indicated that for each unit of increase in PM_2.5_ concentration from preconception to the second trimester, the risk of GDM escalated from 4.2% to 6.7% [21]. PM_2.5_ combined with SO_2_, CO, and O_3_ was substantially correlated with the incidence of GDM. The authors also stated that the risk of GDM was greater in women exposed to both PM_2.5_ and PM_10_, peaking at 61.4% during the second trimester. The risk of GDM progressively escalated with advancing weeks of gestation for PM_2.5_ combined with O_3_ exposure. The highest incidence of GDM during the first trimester was associated with PM_2.5_ plus CO exposure at 8.1%. Lastly, the risk of GDM was linked to PM_2.5_ exposure during the 9 to 11 weeks preceding conception, with the most significant correlation noted at week 11, indicating a 56.6% increased risk of GDM for each 10 mg/m^3^ increase [21].

In a 2023 retrospective study by Celis M.M. et al., the average concentrations of PM_2.5_ and NO_2_ were marginally elevated in women with GDM, although O_3_ levels were comparable between the two groups. The correlations between PM_2.5_ and GDM were the strongest and statistically significant from weeks 7 to 18 of gestation. Elevated PM_2.5_ exposure during early pregnancy and O_3_ exposure in the late first trimester and throughout the second trimester were linked to GDM, while exposure to green spaces may provide a preventive effect [22].

A 2023 cohort study by Papatheodorou et al. was the first study to demonstrate that PM affects pregnancy outcomes such as GDM. The study showed that an increased average PM exposure during the first and second trimesters correlated with elevated chances of GDM for each of the top three quartiles compared to the lowest quartile. Maintaining current EPA restrictions and reducing traffic exposure may mitigate the following adverse effects of PM_2.5_ on morbidity and mortality, as well as radiation exposure and its ensuing consequences [23].

Yan M et al., in 2022, suggested that in comparison to pregnant women without GDM, individuals with GDM exhibited elevated exposure to PM_2.5_, PM_10_, SO_2_, NO_2_, and CO during the first and second trimester, and throughout the entire pregnancy. The authors also suggested that exposure to ambient PM_2.5_ and PM_10_ during the first trimester was strongly correlated with an elevated incidence of GDM [24]. Additionally, the association between PM and the risk of GDM was more significant for PM_2.5_ compared to PM_10_. They showed that ambient PM pollution adversely affects Gestational Hypertension (GH), GDM, and Preeclampsia (PreE) among Chinese pregnant women. Given that numerous areas in China continue to experience perilous levels of air pollution, our findings underscore the necessity of enhancing protections for pregnant women against the risks associated with air pollution. In 2022, Chen X. et al. supported that substantial modifications to the maternal hair metabolome are associated with subsequent GDM development. Three of these modified metabolites were correlated with maternal exposure to air pollution, indicating that environmental contaminants can elevate the incidence of GDM. They suggested that the hair metabolome is modified in reaction to maternal and environmental disturbances, indicating that maternal hair may serve as a means to assess exposure risk factors and enhance comprehension of the underlying pathophysiology of GDM [25].

A study from 2023 by Molitor J. et al., where they used electronic health records from all Kaiser Permanente Southern California (KPSC) facilities, showed that, generally, mothers with GDM, younger mothers, African American or Hispanic mothers with little education, those residing in low-income neighborhoods, and obese mothers had elevated exposure to PM_2.5_ and its chemical constituents. In addition, the overall mass of PM_2.5_ and its constituents, such as NO_3_, organic matter, and black carbon, were elevated among mothers who conceived during the warm season, but PM_2.5_ sulfate levels were increased for mothers who conceived in the cool season [26].

Accordingly, a study conducted by Zhongzheng N. et al. showed that out of 617 participants (78.6% Hispanic and 11.8% Non-Hispanic Black), GDM was identified in 9.7% of the participants that predominately originated from low-income households, with 55% possessing a high school education or below. Of the participants, 67% were overweight or obese before pregnancy and 29% exhibited probable prenatal depression [27]. They suggested that the periconceptional period is a window of susceptibility to ambient PM_2.5_, PM_10_, and NO_2_ exposure with increased risk of GDM. The susceptibility to air pollution concerning the risk of gestational diabetes mellitus (GDM) was elevated among women exhibiting likely prenatal depression, advanced age, or postpartum body mass index (ppBMI) [27].

In a 2019 cohort study by Jo H et al. conducted in Southern California, it was shown that preconception exposure to PM_2.5_ was linked to GDM, but this association significantly decreased when adjusting for the first-trimester PM_2.5_ and either NO_2_ or O_3_ exposure across both exposure periods [28]. Additionally, exposure to PM_2.5_ during the first trimester was linked to a decreased risk of GDM, with this connection being significant in multi-pollutant models that included preconception PM_2.5_ exposure alongside either NO_2_ or P_10_, but not O_3_, in both exposure periods [28].

Shen H.N. et al. conducted a cohort study in Taiwan and noted that elevated pre-pregnancy exposure to PM_2.5_ and SO_2_ was substantially correlated with an increased incidence of GDM. However, they suggested that no significant associations were found between pre-pregnancy exposure to O_3_ or NO_2_ and the incidence of GDM. Lastly, comparable associations were found during the first and second trimesters [29].

In another cohort study conducted in 2015 by Robledo C.A. et al., the authors suggested that maternal exposure to NO_2_ and SO_2_ in the three months preceding conception was linked to an elevated risk of subsequent GDM, whereas preconception exposure to ozone was correlated with a reduced risk of GDM. However, except for sulfate, which correlated with a reduction in GDM risk, preconception maternal exposure to PM_2.5_ constituents did not correlate with GDM [30].

### 4.2. Bisphenol A and Risk for GDM Development

In 2022, Zhu Y. et al. conducted a prospective study that suggested that first-trimester bisphenol S (BPS) concentrations were positively correlated with GDM risk in all individuals, while BPA and triclosan concentrations during early to mid-pregnancy were associated with GDM risk specifically in non-Asians/Pacific Islanders, who exhibit higher urinary phenol concentrations than their Asian/Pacific Islander counterparts. They noted that their discovery of a positive correlation between urine BPS and the risk of GDM may have significant public health implications. In addition, they considered the rising utilization of BPS as an alternative for BPA, and that ongoing biomonitoring and increased awareness of its possible negative health effects are necessary. Lastly, they identified a positive correlation between triclosan exposure during early to mid-pregnancy and the incidence of GDM, necessitating further examination of its impact on further perinatal outcomes [37].

Chen W.J. et al., in 2022, conducted a case–control study and noted that the correlation between BPA and GDM decreased when considering related phenols and parabens, indicating the necessity of examining chemical combinations in perinatal environmental exposure research [38]. In addition, further prospective studies can enhance the comprehension of the association between benzophenone-3 exposure and the development of GDM [38]. Shapiro et al. conducted a cohort study in 2015 and assessed the associations between phthalates, BPA, and metals quantified during the first trimester of pregnancy with impaired glucose tolerance (IGT) and GDM according to established national recommendations [40].

However, no evidence indicates correlations between the newly developing chemicals investigated (phthalates and BPA) and glucose tolerance disorders during pregnancy. But the reliance on a single exposure measurement for these chemicals presents a constraint.

### 4.3. Organophosphorus Flame Retardants and Risk for GDM Development

A case–control study by Lang Q. et al. [35] in 2024 investigated the association between ambient exposure to OPFRs during early gestation and GDM. The research revealed elevated detection rates of OPFRs in women with GDM and their control counterparts, with no significant differences between the two groups. Additionally, tris (1,3-dichloro-2-propyl) phosphate (TDCPP) and tris (2-butoxyethyl) phosphate (TBEP) constituted 74.9% of the overall concentration of OPFRs. It was noted that OPFR levels were markedly higher in women with GDM, indicating a possible association between increased exposure and the onset of GDM. The study by Shapiro G.D. et al. showed no consistent evidence for any positive associations between the chemicals studied and GDM and gestational IGT, and we observed strong inverse associations between dimethyl OP pesticide metabolites and GDM [36].

### 4.4. Extreme Temperatures

In 2021, Huanhuan Z. et al. conducted a cohort study in China and suggested that this study indicated that exposure to extreme high and low temperatures during the second trimester elevates the risk of GDM, with susceptibility windows identified in the 21st to 22nd and 14th to 17th gestational weeks. Additionally, suboptimal temperature serves as an independent factor for GDM [31]. Their findings indicate that clinicians and public health professionals ought to offer health advice to pregnant women, a demographic vulnerable to climate change, particularly regarding the limitation of exposure during extreme temperature events, especially for those in the second trimester of pregnancy [31]. Another study on extreme temperature and its correlation with increased risk for developing GDM by Anais T. et al. proposed that there is an elevated risk of GDM between weeks 20 and 24 of gestation associated with extreme low temperature exposure. Conversely, an elevated risk of GDM was noted during gestational weeks 11 to 16 in association with extreme high temperature exposures [32]. The authors highlighted that since there are specific windows of susceptibility to extreme and low temperatures, we could be able to implement preventative behaviors to mitigate these exposures. In addition, effect modifications were observed through non-normalized difference vegetation index (non-DVI), impervious surface percentage, land surface temperature, water use efficiency, global human settlement, and evapotranspiration canopy. This underscores the potential for implementing interventions that could alter these microclimate indicators, potentially alleviating extreme temperatures and subsequently reducing the risk of GDM [32]. In a 2017 retrospective study by Booth G.L et al., the authors suggested that there is a direct relation between the prevalence of GDM and ambient temperature. The authors revealed that a 10 °C increase in mean 30-day outdoor air temperature corresponded to 1.06 times higher adjusted odds of GDM, assuming screening took place at 27 weeks of gestation. Furthermore, the results exhibited notable similarity when utilizing the mean 30-day outdoor air temperature prior to the established screening test date [33]. A 2015 cohort study conducted in Toronto, Canada, by Retnakaran R. et al. suggested three interesting findings [34]. The authors presented that the change in temperature over the preceding weeks, rather than daily temperature, correlates with blood glucose levels in pregnant women. This association becomes evident during the months of rising temperatures (February–July). Furthermore, the increase in temperature over the 3–4 weeks preceding glucose tolerance testing serves as a significant independent predictor of GDM, even after controlling for clinical risk factors. The fluctuation in temperature during this period is independently linked to beta cell dysfunction, suggesting a possible mechanism by which environmental elements may influence the metabolism of glucose.

### 4.5. Selenium Levels in Urine

Selenium (Se) is a naturally occurring metalloid that is vital for human and animal health in trace quantities; however, it is detrimental in excessive amounts. Se has a significant role in the operation of the human body. It is integrated into selenoproteins, hence bolstering antioxidant defense mechanisms. A study by Liu Y. et al. investigated the relation of urinary Se and the risk of GDM and suggested that there is a notable correlation between reduced urine Se concentration and the incidence of GDM in pregnant women, which remains consistent after adjusting for other potential confounding variables [41]. The correlation between reduced urine Se levels and the incidence of GDM was more significant in pregnant women carrying female fetuses. In another prospective study by Tatsuta N. et al., the authors assessed the correlation between maternal concentrations of metallic elements and GDM in Japanese women [42]. Their findings suggest that exposure to mercury (Hg) and presumably manganese (Mn) during gestation correlates with heightened risk of GDM, but an inverse association exists with whole-blood cadmium (Cd) concentrations, and no correlation is observed with lead (Pb). The concentration of Se in whole blood was correlated with the risk of GDM. Finally, as many metallic elements are acquired through dietary intake, analyzing eating habits, especially during gestation, could be essential for mitigating the risk of GDM.

### 4.6. Urinary Antimony

In a 2019 prospective study by Zhang G. et al., the authors suggested that pregnant women with elevated antinomy (Sb) exposure may have an increased risk of GDM, and this correlation persists even after stratification, indicating that Sb’s significance should be acknowledged in the investigation of GDM pathogenesis [44]. In 2021, Zhang et al. proposed that rubidium (Rb) had a positive correlation with alpha diversity indices, but mercury (Hg) and vanadium (V) demonstrated negative correlations. Additionally, elements such as Rb, thallium (T), arsenic (As), and Sb exhibited significant correlations with GDM-associated core microbiome groups (CAGs). Furthermore, mediation analysis indicated that Rb and Sb were inversely associated with GDM risk by modifying the abundance levels in Lachnospiraceae Coriobacteriales, and Ruminococcaceae [44]. In another prospective study in 2020 conducted in China by Wang et al., the authors highlighted that elevated urine nickel (Ni) concentrations during early pregnancy correlate with an increased risk of GDM, whether assessed separately or as a part of a metal mixture. Moreover, the combined exposure to all six metals, urinary Ni, As, Cd, Sb, cobalt (Co), and vanadium (V), was positively correlated with the incidence of GDM, with Sb and Ni exhibiting more significant effects than the other four metals in the mixture [45]. Lastly, in their 2023 study, Yanyan G. et al. suggested that an increased consumption of thiamine and riboflavin during gestation correlates with a reduced occurrence of GDM [46].

### 4.7. Fetal Sex

As stated from the meta-analysis, (Figure 2), fetal sex is a contributor in the development of GDM (*p* < 0.0001). A higher prevalence has been observed in male fetuses. This can be attributed to the fact that male fetuses have poor β-cell function as well as higher postprandial glycemia, thus increasing the risk of GDM [47]. Complementary to the male sex of the offspring, the age of the mother further increased the risk, as mothers of non-white ethnicity and age greater than 35 had increased likelihood as these were additional aggregating factors [47].

### 4.8. Smoking

Furthermore, the current study did not find a statistical difference (Figure 1) (*p* = 0.8008) in GDM developing between smokers and non-smokers. Additionally, apart from the studies examined in the above meta-analysis, the study by Konstantakou P. further affirms that no correlation has been made between smoking and GDM development; nonetheless, OGTT profile and HbA1c differed according to smoking status in women with and without GDM, allowing room for further investigation [48].

### 4.9. Parity

The meta-analysis performed (Figure 3) revealed a significant association between the number of gestations and development of GDM. The study by Moon J. states that the reason for this can be the fact that multiple pregnancies induce cellular stress in combination with aging features in β-cells, which impairs the proliferative capacity to compensate for the insulin resistance [49].

## 5. Conclusions

Through this meta-analysis, it can be concluded that there is statistical significance for fine particulate matter PM_2.5_, especially in the first (*p* < 0.001) and second (*p* < 0.001) trimesters, proving the acknowledged connection between PM_2.5_ and GDM pathogenesis during pregnancy, while the literature also suggests the cruciality of weeks 9 to 11 pre-conception. Apart from that, fetal sex can play an important role in the development of GDM, as there is greatest risk with males (*p* < 0.001), whereas no correlation between maternal smoking habit and bisphenol A and GDM was found. In conclusion, it can be stated that environmental factors can have a great impact on the development of GDM, during the gestational period, but more studies must be carried out to reinforce our outcomes.

## 6. Limitations

The limitation of this study was that factors such as BMI could not be further examined due to the limited availability of data from the papers examined, but as a thorough examination of the other factors was reported, the outcome of this study was not affected.

## 7. Prospective Investigation

Future studies must be repeated with upcoming data, especially concerning PM_2.5_, an element that women are exposed to daily, especially in large and crowded cities, in addition to OPFRs as fewer related studies were found throughout the search in this study. Additional research can contribute a more detailed understanding of OPFRs’ impact on GDM for additional management measures in terms of both prevention and treatment. Additionally, when it comes to extreme temperatures, this is an issue rising fast, especially with the global warming effect; thus, it is crucial for scientists to pay a closer attention in pregnant women.

## Figures and Tables

**Table 1:** Fine particulate matter (PM_2.5_) as a risk factor.

Author	Population Size	Diagnosed with GDM/ Non-GDM	Mean (SD) Age GDM/ Non-GDM	BMI (SD) GDM/ Non-GDM	Country	Gestational Weeks (SD)	Risk Factor
Wan Z., 2024 [21]	4913	472/4441	N/A	N/A	Guangzhou, China	Pre-conception First trimester Second trimester	Fine particulate matter (PM_2.5_)
Celis M.M., 2023 [22]	1,310,807	68,860/1,241,947	32.72 (5.06)/30.14 (5.43)	28.50 (8.10)/25.71 (7.09)	Toronto, Canada	28.50 (8.10)/25.71 (7.09)	Fine particulate matter (PM_2.5_)
Papatheodorou S., 2020 [23]	1,061,937	43,446/1,018,491	32 (5.5)/-	N/A	Massachusetts, USA	Pre-conception First trimester Second trimester	Fine particulate matter (PM_2.5_)
Yan M., 2022 [24]	3754	420/3334	N/A	N/A	China	Pre-conception First trimester Second trimester	Fine particulate matter (PM_2.5_)
Chen X., 2022 [25]	777	29/748	29.5 (1.25)/28 (1)	24.1 (0.95)/21.05 (0.88)	China	Pre-conception First trimester Second trimester	Environmental pollution, the hair metabolome, fine particulate matter (PM_2.5_)
Molitor J., 2023 [26]	341,909	37,711/304,198	32.56/29.91	N/A	California, USA	First trimester Second trimester	Fine particulate matter (PM_2.5_)
Zhongzheng N., 2023 [27]	617	60/557	31.1 (5.3)/-	N/A	California, USA	Pre-conception First trimester Second trimester	Fine particulate matter (PM_2.5_)
Jo H., 2019 [28]	239,574	18,244/221,330	32.4 (5.4)/29.4 (5.8)	N/A	California, USA	Pre-conception First trimester	Fine particulate matter (PM_2.5_)
Shen H.N., 2017 [29]	13,434	6717/6717	31.30 (4.54)/31.12 (4.510)	N/A	Taiwan	First trimester Second trimester	Fine particulate matter (PM_2.5_)
Robledo C.A., 2015 [30]	219,952	11,334/208,618	N/A	N/A	USA	Pre-conception First trimester	Fine particulate matter (PM_2.5_)

N/A: non-applicable.

**Table 2:** Extreme temperatures as a risk factor.

Author	Population Size	Diagnosed with GDM/Non-GDM	Mean (SD) Age GDM/Non-GDM	BMI (SD) GDM/ Non-GDM	Country	Gestational Weeks (SD)	Risk Factor
Huanhuan Z., 2021 [31]	5421	604/4561	27.55 (4.48)/26.33 (4.02)	N/A	Guangzhou, China	First trimester Second trimester	Extreme temperatures (high; low)
Anais T., 2023 [32]	395,927	42,970/353,957	32.6 (5.3)/30.0 (5.7)	N/A	California, USA	First trimester Second trimester	Extreme temperatures (high; low)
Booth G.L., 2017 [33]	396,828	N/A	N/A	N/A	Toronto, Canada	First trimester Second trimester	Extreme temperatures (high)
Retnakaran R., 2018 [34,35]	1464	318/1146	N/A	N/A	Toronto, Canada	Second trimester	Temperature changes

N/A: non-applicable.

**Table 3:** Organophosphorus Flame Retardants as Risk Factor.

Author	Population Size	Diagnosed with GDM/ Non-GDM	Mean (SD) Age GDM/ Non-GDM	BMI (SD) GDM/ Non-GDM	Country	Gestational Weeks (SD)	Risk Factor
Lang Q., 2024 [35]	150	90/60	33.54 (4.75)/31.36 (4.840)	22.031 (3.40)/21.35 (2.60)	Liuzhou, China	First trimester	Organophosphorus Flame retardants
Shapiro G.D., 2016 [36]	1274	93/1102	N/A	N/A	Canada	First trimester	Organophosphorus Flame retardants

N/A: non-applicable.

**Table 4:** Bisphenol A as a risk factor.

Author	Population Size	Diagnosed with GDM/ Non-GDM	Mean (SD) Age GDM/ Non-GDM	BMI (SD) GDM/Non-GDM	Country	Gestational Weeks (SD)	Risk Factor
Zhu Y., 2022 [37]	333	111/222	31.4 (4.9)/31/1 (4.5)	N/A	California, USA	First trimester Second trimester	Bisphenol A
Chen W.J., 2022 [38]	301	64/237	N/A	N/A	Oklahoma, USA	First trimester	Bisphenol A
Fisher B.G., 2018 [39]	232	47/185	33.1 (4.4)/33.7 (3.8)	N/A	Cambridge, UK	First trimester	Bisphenol A
Shapiro G.D., 2015 [40]	1274	93/1102	N/A	N/A	Canada	First trimester	Bisphenol A

N/A: non-applicable.

**Table 5:** Metals and trace elements as risk factors.

Author	Population Size	Diagnosed with GDM/Non-GDM	Mean (SD) Age GDM/ Non-GDM	BMI (SD) GDM/ Non-GDM	Country	Gestational Weeks (SD)	Risk Factor
Liu Y., 2023 [41]	678	226/452	N/A	N/A	Wuhan, China	Second trimester	Selenium (Se) levels in Urine
Tatsuna N., 2022 [42]	78,964	1623/77,341	33.3 (4.9)/(31.5)	N/A	Japan	Second trimester Third trimester	Metallic element concentrations in the blood (Se, Hg, Cd, Pb, Mn)
Zhang G., 2019 [43]	2093	241/1852	N/A	N/A	Wuhan, China	First trimester	Urinary antimony (Sb)
Zhang Y., 2021 [44]	837	128/709	N/A	N/A	Jiangsu, China	Second trimester	Trace elements (Sb, Cu, Zn, As, Mo, Hg, Tl, Fe, Sr, Ag, V, Co, Rb, Cs)
Wang X., 2020 [45]	2090	241/1849	29.54 (4.13)/28.25 (3.34)	N/A	Wuhan, China	Second trimester	Urinary nickel (Ni), arsenic (As), cadmium (Cd), antimony (Sb), cobalt (Co), vanadium (V)
Yanyan G., 2023 [46]	3036	469/2767	N/A	N/A	Wuhan, China	First trimester Second trimester	Thiamine and riboflavin

N/A: non-applicable.
